# Obituary

**Published:** 1904-11-15

**Authors:** 


### OBITUARY.

---

Dr. George H. Chance, of Portland, Oregon, died September 3, 1904. Dr. Chance was one of the pioneer coast dentists, as well as one of the best known and most highly-esteemed men of that section. He was a genial and accomplished gentleman, and will be mourned by a large circle of friends and acquaintances. He was for a short time connected with the North Pacific Dental College and attended the National Association of Faculties at Niagara Falls, where he showed the greatest interest in the work of the Faculties Association, and made friends of every one he met.

